# Annexin A2 Deficiency Exacerbates Neuroinflammation and Long-Term Neurological Deficits after Traumatic Brain Injury in Mice

**DOI:** 10.3390/ijms20246125

**Published:** 2019-12-05

**Authors:** Ning Liu, Yinghua Jiang, Joon Yong Chung, Yadan Li, Zhanyang Yu, Jeong Woo Kim, Josephine M. Lok, Michael J. Whalen, Xiaoying Wang

**Affiliations:** 1 Clinical Neuroscience Research Center, Department of Neurosurgery, School of Medicine, Tulane University, New Orleans, LA 70112, USA; nliu3@tulane.edu (N.L.); yjiang11@tulane.edu (Y.J.); yli72@tulane.edu (Y.L.); 2 Neuroscience Center, Massachusetts General Hospital, Harvard Medical School, Charlestown, MA 02129, USA; JYCHUNG@PARTNERS.ORG (J.Y.C.); MWHALEN@mgh.harvard.edu (M.J.W.); 3 Neuroprotection Research Laboratory, Massachusetts General Hospital, Harvard Medical School, Charlestown, MA 02129, USA; yu.zhanyang@mgh.harvard.edu (Z.Y.); jeong_woo_kim@brown.edu (J.W.K.); JLOK1@mgh.harvard.edu (J.M.L.)

**Keywords:** traumatic brain injury, Annexin A2, knockout mice, leukocytes infiltration, neuroinflammation, neurological function

## Abstract

Our laboratory and others previously showed that Annexin A2 knockout (A2KO) mice had impaired blood–brain barrier (BBB) development and elevated pro-inflammatory response in macrophages, implying that Annexin A2 (AnxA2) might be one of the key endogenous factors for maintaining homeostasis of the neurovascular unit in the brain. Traumatic brain injury (TBI) is an important cause of disability and mortality worldwide, and neurovascular inflammation plays an important role in the TBI pathophysiology. In the present study, we aimed to test the hypothesis that A2KO promotes pro-inflammatory response in the brain and worsens neurobehavioral outcomes after TBI. TBI was conducted by a controlled cortical impact (CCI) device in mice. Our experimental results showed AnxA2 expression was significantly up-regulated in response to TBI at day three post-TBI. We also found more production of pro-inflammatory cytokines in the A2KO mouse brain, while there was a significant increase of inflammatory adhesion molecules mRNA expression in isolated cerebral micro-vessels of A2KO mice compared with wild-type (WT) mice. Consistently, the A2KO mice brains had a significant increase in leukocyte brain infiltration at two days after TBI. Importantly, A2KO mice had significantly worse sensorimotor and cognitive function deficits up to 28 days after TBI and significantly larger brain tissue loss. Therefore, these results suggested that AnxA2 deficiency results in exacerbated early neurovascular pro-inflammation, which leads to a worse long-term neurologic outcome after TBI.

## 1. Introduction

Pro-inflammatory responses at the acute phase after traumatic brain injury (TBI) are characterized by elevated pro-inflammatory cytokines produced by activated microglia, astrocytes, and infiltrated peripheral inflammatory cells, which cause blood–brain barrier (BBB) permeability and neurotoxicity, thereby worsening outcomes [1,2,3]. Annexin A2 (AnxA2) is a 36 KD multi-compartmental protein that exists as a monomer or a heterotetrameric complex with the S100A10 (P11). Intracellular AnxA2 was proposed to be a key regulator of many biological processes, including exocytosis [4], endocytosis [5], membrane trafficking [6], and apoptosis [7]. Furthermore, extracellular AnxA2 plays a key role in fibrinolysis and anticoagulation [8,9], cell migration [10,11], and angiogenesis [8]. Accumulating data suggests that there is a strong positive correlation between AnxA2 deficiency and inflammation. For example, A2KO mice exhibit enhanced NLRP3-inflammasome activation in dendritic cells [12], elevated inflammatory cytokines (TNF-α, IL-6, IL-1β, and IFN-γ), decreased bacterial clearance by macrophage, and increased superoxide release and neutrophil infiltration in the lung [13,14]. Additionally, a recent study suggested that AnxA2 is an endogenous anti-inflammatory factor that protects the host from excessive inflammatory damage by negatively regulating infection-associated inflammatory response through the TLR4-initiated TRAM–TRIF pathway [15]. Our recent study indicated that A2KO mice displayed blood–brain barrier (BBB) dysfunction, and exogenous administration of recombinant AnxA2 (rA2) protected against hypoxia plus IL-1β-induced cerebral trans-endothelial permeability [16], implying that AnxA2 might be a key endogenous factor that functions in reducing pro-inflammatory response after acute brain injury.

In the present study, we aimed to test the hypothesis that A2KO promotes pro-inflammatory response in the brain and worsens neurobehavioral outcomes after TBI. A2KO mice and matching wild-type (WT) mice were subjected to TBI by using a standard controlled cortical impact (CCI) device. We examined AnxA2 protein expression and inflammatory leukocyte brain infiltration. Expression levels of the pro-inflammatory cytokine and the adhesion molecules in the ipsilateral brain tissue and the isolated micro-vessels were examined and compared in A2KO mice and WT mice after CCI. Furthermore, long-term neurologic outcomes and brain tissue loss after TBI were also determined and compared. Our experimental data suggested increased endogenous AnxA2 in the ipsilateral brain in response to TBI. AnxA2 deficiency results in exacerbated neuroinflammation and neurovascular pro-inflammation, which might be detrimental to long-term neurologic outcomes after TBI. Further investigation is warranted for better understanding of the underlying molecular mechanisms of AnxA2 in TBI pathophysiology and for developing a therapeutic molecular targeting strategy against TBI.

## 2. Results

### 2.1. Upregulation of Endogenous AnxA2 after Traumatic Brain Injury

**Figure 1:**
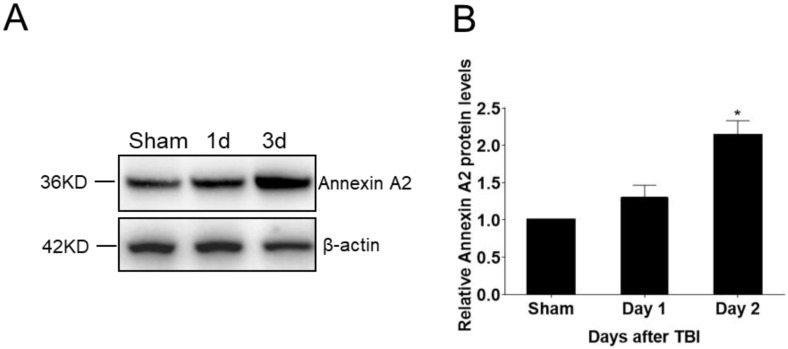
Upregulation of endogenous Annexin A2 (AnxA2) after traumatic brain injury (TBI). Ipsilateral hemisphere of wild-type (WT) mouse brain tissues were collected before and at days one and three after TBI. (**A**) Representative Western blotting images. (**B**) Quantification of AnxA2 protein expression. Data are expressed as mean ± SEM; *n* = 4, * *p* < 0.05 compared to sham.

AnxA2 protein expression in the brain tissue was analyzed before as well as one and three days after TBI. Western blot analysis results showed AnxA2 protein expression was significantly increased at three day post-TBI (Figure 1), indicating a positive response of AnxA2 to TBI.

### 2.2. AnxA2 Deficiency Increases Pro-Inflammatory Cytokine and Adhesion Molecules Expression in the Brain after TBI

**Figure 2:**
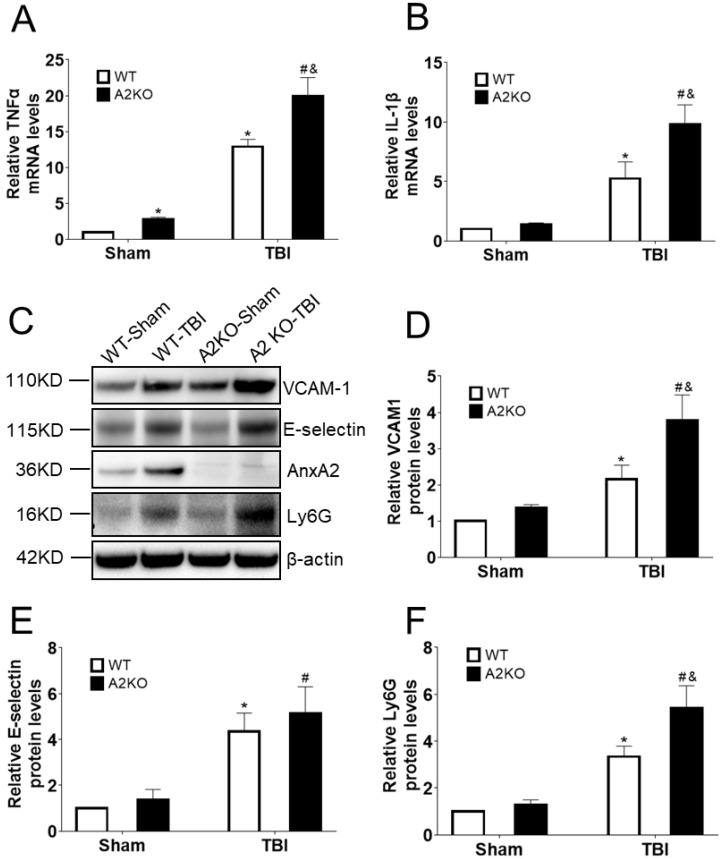
AnxA2 deficiency increases expression of pro-inflammatory cytokine and adhesion molecules in the brain after TBI. (**A**) Quantitative analysis of TNFα mRNA levels in the cortex area of ipsilateral hemisphere in WT and Annexin A2 knockout (A2KO) mice at day one after controlled cortical impact (CCI). (**B**) Quantitative analysis of IL-1β mRNA levels in the cortex area of ipsilateral hemisphere in WT and A2KO mice at day one after CCI. (**C**) Representative gel images of Western blotting for VCAM1, E-selectin, AnxA2, Ly6G, and β-actin expression at day two after CCI. (**D**) Quantification of Western blotting for VCAM1 expression. (**E**) Quantification of Western blotting for E-selectin. (**F**) Quantification of Western blotting for Ly6G. Data are expressed as mean ± SEM; *n* = 4, * *p* < 0.05 compared to WT-sham, ^#^ *p* < 0.05 compared to A2KO-sham, ^&^ *p* < 0.05 compared to WT-CCI.

We next asked whether AnxA2 deficiency might affect neuroinflammatory response in the brain after TBI. Brain tissues were dissected at one day and two days after TBI. RT-qPCR data showed a significantly higher expression of mRNA levels of TNFα and IL-1β in the A2KO mouse brain compared to WT controls at one day after TBI (Figure 2A,B). Moreover, Western blotting analysis indicated that AnxA2 deficiency led to an increase in the protein expression of Ly6G and VCAM1 at two days after TBI (Figure 2C–F). These results suggest that AnxA2 deficiency leads to an elevated pro-inflammatory response in the brain at the early phase after TBI.

### 2.3. AnxA2 Deficiency Increases Cerebral Vascular Pro-Inflammation

**Figure 3:**
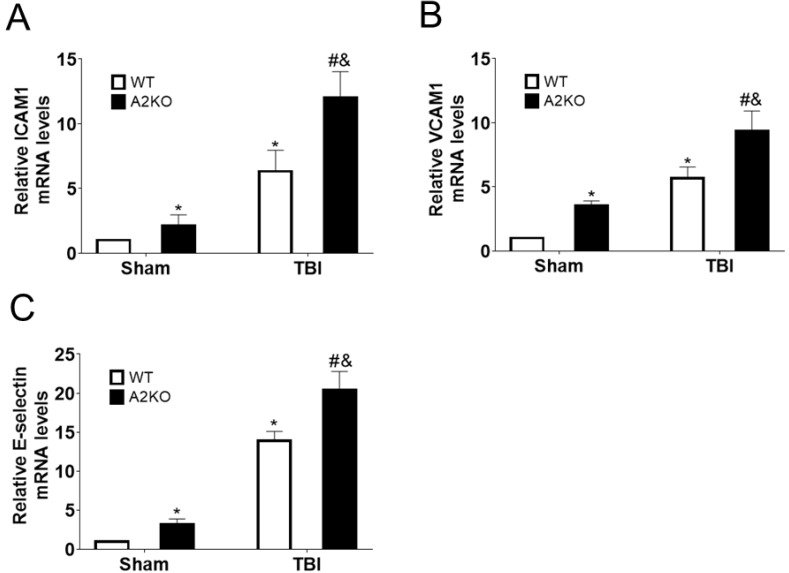
AnxA2 deficiency increases cerebral vascular pro-inflammation after TBI. WT and A2KO mice were subjected to sham injury or CCI, and cerebrovascular fragments were isolated at day one after CCI. (**A**) Quantitative analysis of mRNA levels of ICAM1 expression. (**B**) Quantitative analysis of mRNA levels of VCAM1 expression. (C) Quantitative analysis of mRNA levels of E-selectin expression. Data are expressed as mean ± SEM; *n* = 4, * *p* < 0.05 compared to WT-sham, ^#^ *p* < 0.05 compared to A2KO-sham, ^&^ *p* < 0.05 compared to WT-CCI.

To investigate the potential role of AnxA2 in vascular inflammation, mRNA was extracted from isolated cortical micro-vessels at the peri-lesion area of WT and A2KO mouse brains at day one after TBI. RT-qPCR data showed mRNA expression levels of vascular adhesion molecules ICAM1, VCAM1, and E-selectin were all significantly increased in the A2KO mice compared to the WT controls after TBI (Figure 3), indicating that AnxA2 deficiency leads to an increased vascular inflammation.

### 2.4. AnxA2 Deficiency Enhances Leukocytes Brain Infiltration after TBI

**Figure 4:**
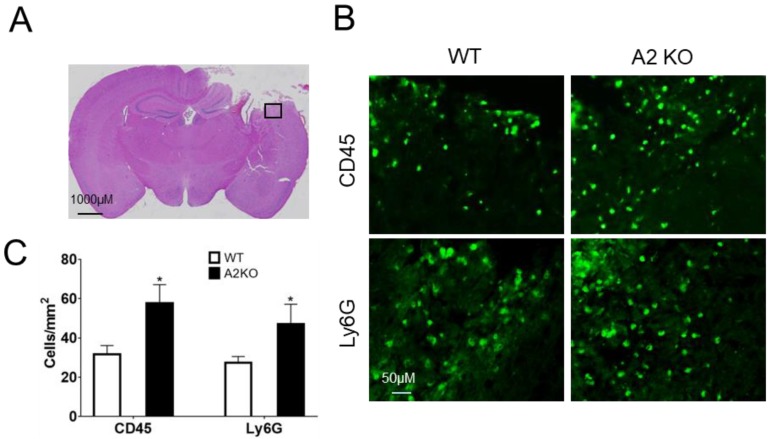
AnxA2 deficiency enhances leukocytes brain infiltration after TBI. Brain tissues were collected at day two after TBI and sectioned for hematoxylin and eosin (H&E) staining and immunohistochemistry. (**A**) The black box in the representative H&E staining indicates the image field of CD45 and Ly6G-immunestaining in ipsilaternal cortex. (**B**) Representative CD45 and Ly6G-immunestaining images. (**C**) Quantitation of CD45 and Ly6G-positive cells in peri-lesion cortex of ipsilateral hemisphere. Data are expressed as mean ± SEM; *n* = 4, * *p* < 0.05 compared to WT.

To investigate potential effects of A2KO in leukocytes brain infiltration after TBI, immunostaining was performed at two days after TBI. Our results showed a significant increase of leukocyte marker CD45-labeled cells and neutrophil marker Ly6G-labeled cells on the cerebral cortical sections of A2KO mouse brains compared to the WT control mice (Figure 4). These results suggest that AnxA2 deficiency exacerbates leukocyte brain infiltration after TBI.

### 2.5. AnxA2 Deficiency Worsens Neurological Outcomes after TBI

**Figure 5:**
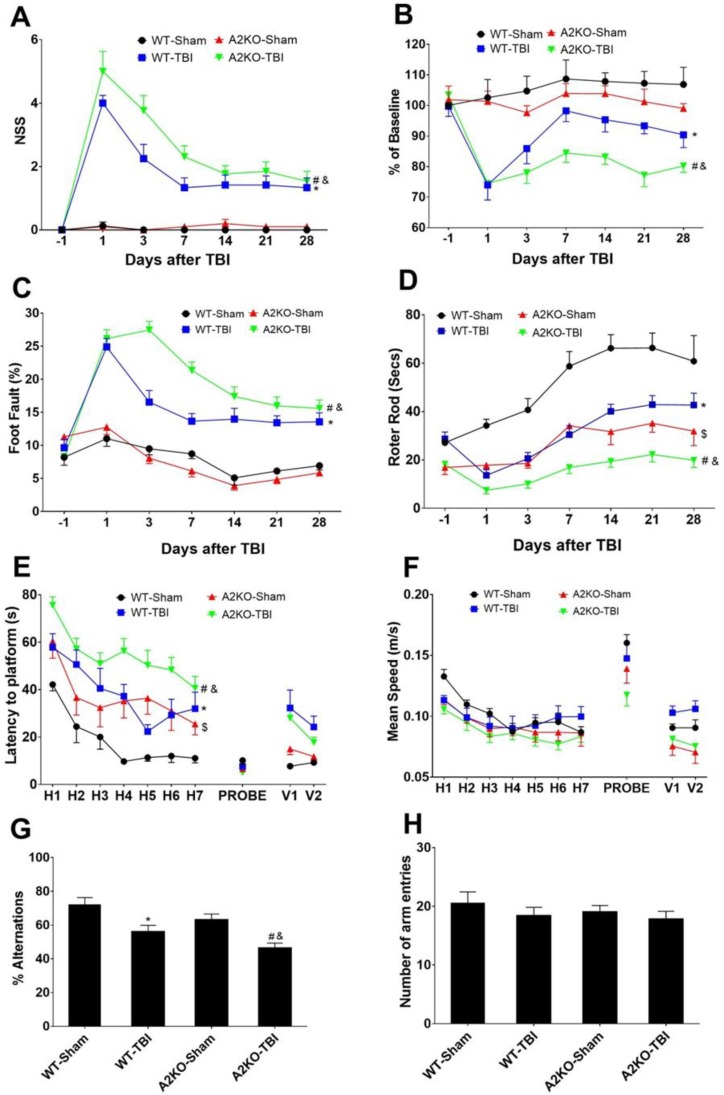
AnxA2 deficiency worsens neurological outcomes after TBI. WT and A2KO mice were subjected to sham injury or TBI. (**A**) Modified neurological severity score (NSS). (**B**) Grip test. (**C**) Food fault test. (**D**) Rota Rod test. (**E**,**F**) Morris Water maze. (**G**,**H**) Y-maze test was performed to assess neurobehavioral outcome up to 28 days after TBI. Sample sizes were WT-sham, *n* = 8; WT-TBI, *n* = 12; A2KO-sham, *n* = 10; A2KO-TBI, *n* = 13, respectively. Data are expressed as mean ± SEM; * *p* < 0.05 compared to WT-sham, ^#^ *p* < 0.05 compared to A2KO-sham, ^&^ *p* < 0.05 compared to WT-TBI, ^$^ *p* < 0.05 compared to WT-sham.

To evaluate the potential role of AnxA2 in neurological outcomes after TBI, we performed neurobehavioral assessments and compared between WT and A2KO mice before and at post-TBI days one, three, seven, 14, 21, and 28. Neurological severity score, grip strength, foot fault, and Rota Rod tests were used to determine sensorimotor functions. Compared to WT mice, A2KO mice had significantly worse functional deficits in all sensorimotor function tests (Figure 5A–D). Morris Water maze and Y-maze tests were used to examine cognitive function deficits after TBI. Morris water maze results showed that A2KO-TBI mice spent a significantly longer time finding the platform compared to the WT-TBI control mice, indicating worse spatial learning and memory deficit in A2KO mice after TBI (Figure 5E), but no difference in speed between groups was found (Figure 5F). Consistently, the Y-maze test showed that, compared to WT-TBI mice, A2KO-TBI mice had lower alternation ratio (Figure 5G), but there was no difference in the total number of arm entries (Figure 5H), suggesting a significantly worse working memory deficit in A2KO-TBI mice. Taken together, these results demonstrate that A2KO worsens neurological deficits in both sensorimotor and cognitive functions compared to the WT mice after TBI.

### 2.6. AnxA2 Deficiency Worsens Brain Tissue Loss after TBI

**Figure 6:**
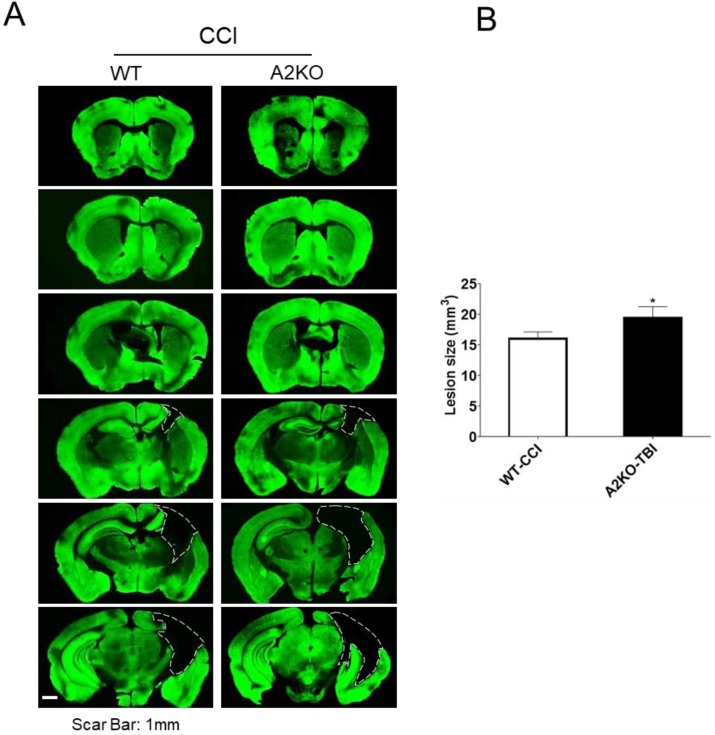
AnxA2 deficiency worsens brain tissue loss after TBI. WT and A2KO mice were subjected to sham injury or TBI. (**A**) Six coronal sections spanning from 1.54 mm anterior to Bregma (Br +1.54 mm) to 3.46 mm posterior to Bregma (Br −3.46 mm) were stained with MAP2 at 29 days post-TBI. The dashed line indicates the border of the brain tissue loss. Scar bar, 1 mm. (**B**) Quantification of brain tissue loss. Data are expressed as mean ± SEM; *n* = 6 per group. * *p* < 0.05 compared to WT-TBI.

Lastly, at 29 days after TBI, brains were removed followed by sectioning. The volume of brain tissue loss was quantitated in MAP2 stained sections. Our result showed that A2KO had significantly larger brain tissue loss compared to WT mice (21% increase) (Figure 6), indicating that A2KO worsens brain tissue damage compared to WT mice after TBI.

## 3. Discussion

The major experimental findings of the present study can be summarized: (1) AnxA2 expression was significantly up-regulated in response to TBI; (2) compared with WT mice, A2KO mice displayed a significant increase of leukocyte brain infiltration and expression for pro-inflammatory cytokines and adhesion molecules in the ipsilateral brain after TBI; (3) the A2KO mice had significantly worse neurological function deficits and increased brain tissue loss compared to the WT controls after TBI. These results indicate a potentially important role of AnxA2 in maintaining brain function homeostasis and protecting against traumatic brain injury.

We found a significant increase of AnxA2 expression at three days after TBI in the ipsilateral brain. Consistently, an earlier study reported that AnxA2 expression was increased after traumatic spinal cord injury [17]. The most important finding of the present study is the association of AnxA2 deficiency with the increased leukocyte brain infiltration, which is consistent with previous studies showing that A2KO mice exhibit increased pulmonary neutrophil infiltration in polymicrobial sepsis models [14] as well as in response to subacute alveolar hypoxia [18] compared to WT mice. Two potential mechanisms are underlying the role of AnxA2 in regulating leukocytes brain infiltration. Firstly, it is well known that leukocytes adhesion to the vascular endothelium wall by adhesion molecules is the prerequisite for the process of leukocytes penetration crossing the blood vessel [19,20]. We observed a significant increase of adhesion molecule expression for ICAM1, VCAM1, and E-selectin in the isolated brain micro-vessels of A2KO mice after TBI compared to the WT mice. These studies indicated that AnxA2 might play key roles in regulating expression and spatial distribution of endothelial adhesion molecules. Secondly, the increased leukocytes infiltration might also be at least partially attributed by the BBB integrity disruption in the A2KO mice. There were previous reports of increased pulmonary neutrophil infiltration in A2KO mice during alveolar hypoxia, which is associated with a reduced endothelial barrier function compared to the WT mice [18]. Moreover, we recently reported that A2KO mice compromise brain microvascular integrity [16]. Therefore, the BBB dysfunction in the A2KO mice might also contribute to the potentiated brain infiltration of inflammatory leukocytes in the A2KO mouse brain after TBI.

In this study, we also found that A2KO manifest an increased pro-inflammatory response with a significantly higher expression of pro-inflammatory TNFα and IL-1β mRNA in the A2KO mouse brain compared with the WT mice after TBI. The increased brain infiltration of inflammatory leukocytes is likely one of the underlying mechanisms. Interestingly, a previous study demonstrated that AnxA2 deficiency led to an intensified pro-inflammatory response associated with an increased oxidative burst and inflammatory response after bacterial infection in the lung [13]. Importantly, another study showed that AnxA2 exerted an anti-inflammatory role in pulmonary infiltrated neutrophils and monocytes by inhibiting reactive oxygen species (ROS) and IL-17 signaling [14]. Taken together, AnxA2 deficiency may lead to elevation of leukocyte brain infiltration and might also enhance ROS and IL-17 signaling in the infiltrated leukocytes and resident microglia, thereby exacerbating the pro-inflammatory response in the brain after TBI.

Likely, the increased leukocyte brain infiltration and the pro-inflammatory cytokine expression in the A2KO mouse brains at the early phase after TBI contribute to the increased brain tissue loss and the worsened neurological function deficits. It is also worth noting that there were significant differences in the pre-TBI baseline levels in the grip and the Rotor Rod tests between WT and A2KO sham groups. The poorer performance of motor function in A2KO mice was speculated upon by a recent study, which reported that A2KO mice had a mild but progressive muscle weakness in locomotor activity [21]. Therefore, the decline of muscle strength or the worse locomotor activity of A2KO mice might also contribute to the worse sensorimotor function deficits after TBI. The baseline difference of neurological function and underlying neurobiological mechanisms need to be further investigated and elucidated in the future.

Although our experimental findings suggest an important role of endogenous AnxA2 for maintaining neurovascular unit integrity and protecting against TBI, several limitations still exist. First, we are aware that A2KO mice may have potential developmental deficits, such as BBB integrity and muscle strength. To overcome this limitation, AnxA2 conditional and tissue-specific knockout mice should be used in future studies. Second, this is a pilot study to investigate the association of Anxn2 with brain inflammation, but the potential roles and mechanisms of endogenous AnxA2 in TBI-induced neuroinflammation remain largely unknown. More detailed information on the effects of AnxA2 deficiency in brain inflammation and underlying molecular mechanisms need to be further elucidated. Third, since AnxA2 is widely expressed in neurons [22,23,24], endothelial cells [25], astrocytes [25], and microglia [26], the biological role of AnxA2 in each brain cell type and the functional couplings between components of neurovascular units in response to TBI warrant investigations. Fourth, further investigations are still needed to test whether AnxA2 is a potentially therapeutic target for post-TBI treatment. We recently showed that recombinant AnxA2 (rA2) could protect against hypoxia plus IL-1β-induced cerebral trans-endothelial permeability in vitro [16], implying that rA2 might be a cerebrovascular targeting agent that could therapeutically protect against BBB leakage and cerebrovascular pro-inflammatory reaction after TBI.

## 4. Materials and Methods

### 4.1. Animals

All animal experiments were carried out following a protocol approved by the Institutional Animal Care and Use Committee of both the Massachusetts General Hospital (#2014N000332 on 11 March 2015) and the Tulane University School of Medicine (#845 on 30 September 2019) under the National Institutes of Health Guide for Care and Use of Laboratory Animals. C57 BL/6 mice were purchased from Charles River Laboratories. A2KO mice on the C57 BL/6 background were generated as described previously [8]. All experiments were performed with randomization, allocation concealment, and blinding.

### 4.2. Controlled Cortical Impact Model of Traumatic Brain Injury

The controlled cortical impact (CCI) model was used as we previously described with a slight modification [27,28,29]. Male A2KO mice and matching wild-type control mice (C57BL/6, 12–14 weeks) were anesthetized with 2% isoflurane (Anaquest, Memphis, TN, USA) in 70% N_2_O and 30% O_2_ using a Fluotec 3 vaporizer (Colonial Medical, Amherst, NH, USA) and fixed into a stereotaxic apparatus. The scalp was opened, and a 5 mm burr hole was made on the left cerebral hemisphere between the bregma and the lambda by using a portable trephine drill (Fine Science Tools, Foster City, CA, USA). Subsequently, the controlled cortical impact was established by a pneumatic cylinder with a 3 mm flat-tip impounder (6 m/s impact velocity, 0.6 mm impact depth, and 100 ms duration). Then, the scalp was sutured immediately following injury, and mice were allowed to recover in the cages. The duration of the entire surgical procedure was about 10 min. Sham control mice were subjected to the identical procedure without cortical impact.

### 4.3. Brain Microvessels Preparation

Brain micro-vessels were prepared as we described previously [30]. Briefly, mice were sacrificed at 24 h after TBI and perfused transcardially with ice-cold PBS. Ipsilateral hemispheres were separated. The cerebellum and the underlying white matter were removed, and then further rolling on the filter paper was performed to remove big vessels. Collected cortical tissues from each hemisphere were homogenized with ice-cold PBS (1 mL for 1 mg of tissue) on ice with Knote Dounce glass tissue grinder (Part 885300-0002; Kimble Chase Life Science, Vineland, NJ, USA) and further centrifuged at 4 °C, 1000× *g* for 5 min. Subsequently, the pellet was re-suspended with 18% Dextran solution (molecular weight 60–90 kDa; USB Corporation, Cleveland, OH, USA) in PBS and then centrifuged again at 4 °C, 1500 g for 20 min. The new pellet was further washed once in PBS and filtered through a 40 μm cell strainer to get rid of the debris. The micro-vessels on the top of the cell strainer were used directly for RNA extraction.

### 4.4. Real-Time Quantitative PCR (RT-qPCR)

RT-qPCR was performed as we described previously [31]. Since it is known that elevated pro-inflammatory cytokine gene expression occurs from hours to days after TBI [32], total RNA from mouse cortical brain tissues or micro-vessels were isolated at day 1 after TBI with miRNeasy micro kit (Qiagen, Germantown, MD, USA) according to the manufacturer’s instruction followed by cDNA synthesis with QuantiTect Reverse Transcription Kit (Qiagen). The mRNA levels were measured by RT-qPCR using TaqMan^®^ Fast Advanced Master Mix (Applied Biosystems, Foster City, CA, USA) in an ABI 7500 real-time PCR system (Applied Biosystems). TaqMan probes were used as follows: Mm00434228_m1 (IL-1β), Mm00443258_m1 (TNFα), Mm00516024_g1 (ICAM1), Mm01320970_m1 (VCAM1), Mm00441278_m1 (E-selectin), Mm01545399_m1 (HPRT). Changes in gene expression (fold change) were determined using the 2^−ΔΔ*C*t^ method with normalization to HPRT. RT-qPCR was performed in triplicate for each sample.

### 4.5. Western Blotting

Brain samples were homogenized in cell lysis buffer (cell signaling) with protease inhibitors (Thermo Fisher Scientific, Waltham, MA, USA). Western blotting (WB) was performed as we previously described [33]. After quantification with the Bradford method, 30 μg protein samples were loaded to 4–12% NuPAGE gel for electrophoresis and subsequently transferred on to PVDF membrane. After blocking with 5% fat-free milk, the membranes were incubated with primary antibodies at 4 °C overnight. Information for the primary antibody is listed as the following: VCAM-1 antibody (E-10) (1:500, Santa Cruz, sc-13160), E-Selectin Antibody (D-7) (1:500, Santa Cruz, sc-137054), AnxA2 antibody (1:500, Santa Cruz, sc-28385), Ly6G antibody (1:300, BD Pharmingen, 550291), and β-actin antibody (1:3000, Sigma, A5441). Subsequently, the membranes were washed with TBST [tris-buffered saline (250 mM Tris, 27 mM KCl and 1.37 M NaCl, pH 7.4) containing 0.1% Tween 20] and further incubated with horseradish peroxidase-conjugated secondary antibodies for 1 h. Enhanced chemiluminescence (GE Healthcare) was performed, and images were captured with G-BOX (Syngene, Frederick, MD, USA). Quantification of protein band intensity obtained by Western blotting analysis was analyzed with Image J (http://rsb.info.nih.gov/ij/) and normalized to the actin band intensity.

### 4.6. Hematoxylin and Eosin Staining

At 2 days after TBI, coronal brain sections were stained with hematoxylin and eosin (H&E) as we previously described [33]. Briefly, mouse brains were collected and post-fixed with 4% paraformaldehyde (PFA) at 4 °C overnight. Brain sections were cut using a Leica VT1000M vibratome (Leica, Buffalo Grove, IL, USA) and attached on Superfrost Plus slide (Menzel-Glaser, Braunschweig, Lower Saxony, Germany). Sections were stained with hematoxylin for 5 min to stain nuclei and washed for 3 min with water. Next, sections were stained with eosin for 3 min followed by rinsing for 1 min. The sections were further dehydrated with 70% ethanol, 100% ethanol, and 100% ethanol (in respective order) for 10–15 s each. Then, the sections were cleared using xylene and photographed.

### 4.7. Immunostaining

Immunostaining was performed as we previously described [33,34]. Since it was reported that brain infiltration of circulating leukocytes after TBI in mice is significantly increased with peak at 1–3 days [35], the infiltrated leukocytes were determined at day 2 after CCI. After anesthetization with 2% isoflurane, the mice were perfused transcardially with ice-cold PBS followed by 4% paraformaldehyde in phosphate buffered saline. Brains were removed and post-fixed for 24 h in 4% PFA at 4 °C and transferred into 30% sucrose at 4 °C for 3 days. The brain tissues were sliced into 16 µm-thick coronal sections using a microtome (Leica, Buffalo Grove, IL, USA). After blocking in PBS with 5% bovine serum albumin (BSA) for 1 h at room temperature, the brain sections were incubated at 4 °C overnight in a blocking buffer containing the primary antibodies. The primary antibody information is as follows: CD45 antibody (1:100, 550539, BD Pharmingen, San Jose, CA, USA), Ly-6G and Ly-6C antibody (1:100, BD Pharmingen, 550291). Subsequently, the sections were washed with PBS and further incubated with secondary antibody conjugated to fluorescein (1:250, Thermo Fisher Scientific, Waltham, MA, USA) for 1 h at room temperature followed by washing with PBS and staining with DAPI. Fluorescence signals in the peri-lesion cortex were captured using an Olympus inverted microscope (BX51; Olympus, New Orleans, LA, USA). For quantitative analyses, observers were blinded to the experimental groups. The number of positive stained cells per 0.20 square millimeter (0.5 mm × 0.4 mm; 20× magnification) were counted in four consecutive selected microscopic fields in the peri-lesion cortex from the third brain slice reflecting the maximal change of lesion size between groups as we previously described [36]. The average cell number was calculated for each experimental group.

### 4.8. Assessments of Sensorimotor Function Deficits

We assessed sensorimotor neurobehavioral functions on days 1, 3, 7, 14, 21, and 28 as we described previously [27,28,29,37]. Neurological Severity Score (NSS): a 10-point NSS was used to quantify motor function, and one score point was awarded for the inability to perform each of the tasks as previously described [27]. Grip strength test: a grip strength test was performed as we previously described [33]. The results were quantified as percentage of each individual pre-injury baseline. Grid walk test: a grid walk test was performed as we previously described [27,33]. The grid containing 11 ×11 mm openings was used. Front ipsilateral foot faults and total frontal feet steps were counted within 1 min. The foot that went through the grid opening was considered as a foot fault. Rota Rod test: a Rota Rod test was performed to evaluate sensorimotor balance and coordination as we previously described with minor modification [37]. Mice were placed on an accelerating rotating drum (Harvard Apparatus, Holliston, MA, USA) from 4 to 40 rpm for 5 min until they dropped from the rod, and the total running time spent on the device was automatically recorded. Mice were tested four times with an inter-trial interval of 20 min. The average latency of the four trials was calculated. Mice were trained for 2 days, and the baseline was obtained at the day before surgery.

### 4.9. Assessments of Cognitive Function Deficits

Y-Maze: a spontaneous alternative Y-maze test was conducted on day 20 after CCI for analyzing spatial working memory as we described previously [33,38]. The Y-maze apparatus was made of solid gray plastic and consisted of three arms (395 mm in length, 120 mm in height, at 120° angles to each arm). Mice were placed in the center of the maze and were allowed to explore the maze for 5 min, which was captured by a digital video. The number of arm entries and the number of triads (a set of consecutive arm entries, e.g., triplets of ABC, BCA, CAB, etc.) were counted to calculate the alternation percentage. The ratio of correct choice was determined by the equation: % alternations = [(number of alternations)/(total arm entries − 2)] × 100). Morris water maze (MWM): a MWM test was conducted to evaluate spatial learning from days 10 to 14 after CCI as we previously described [27,28]. Mice were given five hidden platform trials (one to two trials per day), a probe trial, and visible trials 24 h after the hidden platform trials.

### 4.10. Quantitation of Traumatic Brain Tissue Loss

Quantitation of traumatic brain tissue loss was performed as described previously [39]. Briefly, six equally-spaced slices from bregma: +1.54 to −3.46, with 1000 μm interval. Coronal brain sections were stained with Alexa Fluor^®^ 488 conjugated MAP2 antibody (1:200, MAB3418X, Sigma-Millipore, Burlington, MA, USA), and the brain tissue loss was quantified with Image J software and calculated with the following formula: V_C_–V_L_ (V_C_ is the volume of the contralateral hemisphere, V_L_ is the volume of residual tissue in the ipsilateral hemisphere).

### 4.11. Statistical Analysis

Statistical analyses between multiple groups were analyzed by one-way or two-way ANOVA followed by Tukey post hoc test using GraphPad Prism 7 software (San Diego, CA, USA). Single comparisons were evaluated by paired *t*-test. All data were expressed as mean ± SEM. Differences were considered statistically significant at *p* < 0.05.

## 5. Conclusions

In summary, the present study showed AnxA2 deficiency led to exacerbated neuroinflammation and neurovascular pro-inflammation, which might be detrimental to long-term neurologic outcomes after TBI. Further investigation is warranted for better understanding of the underlying molecular mechanisms of AnxA2 in TBI pathophysiology and for developing a therapeutic molecular targeting strategy against TBI.

## Abbreviations

TBI	Traumatic brain injury
CCI	Controlled cortical impact
WT	wildtype
A2KO	Annexin A2 knockout
AnxA2	Annexin A2
BBB	blood-brain barrier
WB	Western Blotting
RT-qPCR	Real-time quantitative polymerase chain reaction
IL-1β	Interleukin 1 beta
TNFα	Tumor necrosis factor-alpha
